# Crying as a precipitating factor for migraine and tension-type headache

**DOI:** 10.1590/S1516-31802003000100008

**Published:** 2003-01-02

**Authors:** Yara Dadalti Fragoso, Regina Carvalho, Fernanda Ferrero, Darcya Maria Lourenço, Erica Regina Paulino

**Keywords:** Headache, Migraine, Tension-type, Crying, Cefaléia, Enxaqueca, Tipo, Tensional, Choro

## Abstract

**CONTEXT:**

Scarcely reported in the literature, crying seems to be an important precipitating factor for both migraine and tension-type headache in daily practice.

**OBJECTIVE:**

To evaluate the role of crying as a precipitating factor for migraine and tension-type headache.

**TYPE OF STUDY:**

Prospective evaluation.

**PARTICIPANTS:**

163 workers or students from the Universidade Metropolitana de Santos, who presented at least one attack a month, for at least one year, of either migraine or tension-type headache.

**PROCEDURES:**

Interview by means of questionnaires and personal evaluations. Details of precipitating factors for the attacks were assessed.

**RESULTS:**

From the total group of 163 individuals, 90 (55.2%) considered crying to be a potential factor for triggering headache attacks. Of this group of 90 persons, 62 presented migraine (6 males, 56 females) and 28 presented tension-type headache (5 males, 23 females). Only stress, anxiety and menstrual periods rated higher or equal to crying as triggering factors for both types of headache.

**CONCLUSIONS:**

The physiology of crying is not well documented or understood. The act of crying seems to be an important precipitating factor for primary headaches and it should be studied further. The authors welcome comments on the matter and would like to work in collaboration with other groups interested in this subject.

## RESUMO

**CONTEXTO:**

Pouco relatado na literatura, o ato de chorar parece ser um importante fator precipitante de enxaqueca e cefaléia do tipo tensional na prática clínica diária.

**OBJETIVO:**

Avaliar o papel do choro como fator precipitante de enxaqueca e cefaléia do tipo tensional.

**TIPO DE ESTUDO:**

Avaliação prospectiva.

**PARTICIPANTES:**

163 funcionários ou estudantes da Universidade Metropolitana de Santos que apresentavam cefaléia pelo menos uma vez por mês, por pelo menos um ano, sendo o diagnóstico enxaqueca ou cefaléia do tipo tensional.

**PROCEDIMENTOS:**

Entrevista usando questionário e avaliação individual. Detalhes dos fatores precipitantes das crises foram avaliados.

**RESULTADOS:**

Do grupo de 163 funcionários ou estudantes, 90 (55.2%) consideravam o choro como um potencial desencadeante de crises de cefaléia. Deste grupo de 90 pessoas, 62 apresentavam enxaqueca (6 homens e 56 mulheres) e 28 apresentavam cefaléia do tipo tensional (5 homens e 23 mulheres). Apenas estresse, ansiedade e período menstrual foram tão ou mais importantes que o choro como desencadeantes dos dois tipos de cefaléia.

**CONCLUSÕES:**

A fisiologia do choro não é bem documentada ou compreendida. O ato de chorar parece ser um importante fator desencadeante de cefaléias primárias e deveria ser estudado em maior detalhe. Os autores gostariam de comentários neste tema e gostariam também de trabalhar em colaboração com outros grupos interessados no tema.

## INTRODUCTION

A variety of precipitating factors have been implicated in triggering migraine attacks. These factors, sometimes spontaneously mentioned by the patients during the consultations, have to be carefully investigated during the anamnesis. Details of attacks triggered by menstrual periods, strong light or smell, noises, certain foods and sudden weather changes, just to mention a few, are often asked about by the headache specialist.

However, the act of crying, which seemed to the authors to be a particularly common triggering factor, is not usually asked about or noted in the patient's files. Similarly, the literature is scarce when we consider "crying" as a precipitating factor for attacks. There are few articles reporting on "crying migraine"^1,2^ and none reporting on "crying tension-type headache".

We have therefore evaluated a population of individuals suffering from migraine or tension-type headache, attempting to access the role of crying as a precipitating factor. Whenever the individual presented both types of headache, we asked them to consider the one that was most frequent and distressing. If the headache could not be clearly classified, the person was excluded. The criteria of the International Headache Society were used for classifying the headache under investigation.^3^ Workers and students at the Universidade Metropolitana de Santos were recruited to participate in this study when they presented headache attacks at least once a month for at least one year. A group of 163 individuals from these workers and students was selected by means of a questionnaire and personal interviews.

Throughout the evaluation, the individuals were kept unaware of our interest in crying as a possible precipitating factor. The questions about crying details were included among several other questions and no particular emphasis was placed on crying as a potential triggering factor. From a list of possible situations and factors that could precipitate an attack, the individual was asked to identify those, which, in his/her particular case, had been clearly responsible for an attack, at least once in his/her life.

Demographic data and results are presented in the Table. In summary, 21 males and 142 females entered this study. The average age was 25 years (ranging from 16 to 70). Educational level varied from basic literacy to postgraduate lecturers. A subgroup of 75 individuals (46%) presented migraine while the remaining 88 (54%) presented tension-type headache. From the total group of 163 individuals, 90 (55.2%) considered crying as a potential factor for triggering attacks of headaches. Of this group of 90 persons, 62 presented migraine (6 males, 56 females) and 28 presented tension-type headache (5 males, 23 females).

**Table:** Summarized results concerning precipitating factors of migraine and tension-type headache (TTH) in a group of individuals presenting at least one headache attack per month for at least one year. Patients (n = 163), 21 men and 142 women, with average age of 25 years

	Migraine	TTH
Precipitating factors*	75 (46%)	88 (54%)
Stress/anxiety	73	52
Weather changes	28	20
Alcohol	25	21
Menstrual periods	52(out of 56 females)	19 (out of 58 females)
Light/brightness	46	21
Odors	46	17
Crying	62 (82.6%)	28 (53.8%)
– Personal reasons	55 (88.7%)	26 (92.8%)
– Sadness only	18 (29.1%)	16 (57.1%)
– Sadness or happiness	44 (70.9%)	10 (35.7%)
– Other reasons	15 (24.1%)	0
– Cannot specify details	3 (4.8%)	2 (7.1%)

* *(may be more than one per patient).*

For both migraine and tension-type headache, prolonged anxiety and stressful situations were considered to be the most important triggering factors, almost invariably leading to a headache attack (76.6% of all the 163 cases). Crying (55.2%) and menstruation (54.2% of all women) also rated high as possible precipitating factors. Other situations, such as exposure to light, smell, sound, weather changes, and alcohol were also mentioned, though none as frequently as the above-cited stress, crying and menstruation.

While further investigating the details of triggering factors, it was interesting to notice the reasons for crying that could precipitate an attack. Migraine patients mentioned that attacks could be induced by crying caused by personal reasons (88.7%), sadness only (29.1%), sadness or happiness (70.9%) or even external reasons, such as a romantic movie (24.1%). Meanwhile, tension-type headache patients referred to headache attacks induced by crying caused by personal reasons (92.8%), sadness only (51.7%) and sadness or happiness (35.7%). No individual with tension-type headache mentioned crying because of external situations as a possible precipitating factor. Only five persons (three migraine and two tension-type headache) could not give further details about crying and headache. These persons merely observed that this could be a precipitating factor but were not sure of the details. Interestingly, all patients seemed to understand that headaches triggered by stress and anxiety involved different precipitating factors from the crying itself, even if the reason for crying came from the stress and/or anxiety.

In summary, and as reported by Evans, crying may be a common under-recognized migraine trigger.^2^ In the present report, we suggest that crying may precipitate both migraine and tension-type headache and, if carefully investigated, a high percentage of patients will be aware of details of such a trigger factor. The physiology of crying is not well documented and perhaps neuro-transmission and increased intracranial pressure may suffice to explain the role of crying in triggering primary headache attacks. We would like to bring out this matter for discussion and to participate in other studies concerning this subject.
